# Novel Scheme for Defining the Clinical Implications of *TP53* Mutations in Myeloid Neoplasia

**DOI:** 10.21203/rs.3.rs-2656206/v1

**Published:** 2023-03-09

**Authors:** Waled Bahaj, Tariq Kewan, Carmelo Gurnari, Arda Durmaz, Ben Ponvilawan, Ishani Pandit, Yasuo Kubota, Olisaemeka D. Ogbue, Misam Zawit, Yazan Madanat, Taha Bat, Suresh K. Balasubramanian, Hussein Awada, Ramsha Ahmed, Minako Mori, Manja Meggendorfer, Torsten Haferlach, Valeria Visconte, Jaroslaw P. Maciejewski

**Affiliations:** Cleveland Clinic; Cleveland Clinic; Cleveland Clinic; Cleveland Clinic; Cleveland Clinic; Cleveland Clinic; Cleveland Clinic; Cleveland Clinic; Cleveland Clinic; University of Texas Southwestern Medical Center; University of Texas Southwestern Medical Center; Wayne State University; Cleveland Clinic; Cleveland Clinic; Cleveland Clinic; MLL Munich Leukemia Laboratory; MLL Munich Leukemia Laboratory; Cleveland Clinic; Cleveland Clinic

**Keywords:** TP53 mutations, allelic inactivation, myeloid neoplasia, next generation sequencing, single-cell DNA sequencing

## Abstract

**Background:**

*TP53* mutations (*TP53*^*MT*^) occur in diverse genomic configurations. Particularly, biallelic inactivation is associated with poor overall survival in cancer. Lesions affecting only one allele might not be directly leukemogenic, questioning the presence of cryptic biallelic subclones in cases with dismal prognosis.

**Methods:**

We have collected clinical and molecular data of 7400 patients with myeloid neoplasms and applied a novel model to properly resolve the allelic configuration of *TP53*^*MT*^ and assess prognosis more precisely.

**Results:**

Overall, *TP53*^*MT*^ were found in 1010 patients. Following the traditional criteria, 36% of cases were classified as single hits while 64% exhibited double hits genomic configuration. Using a newly developed molecular algorithm, we found that 579 (57%) patients had unequivocally biallelic, 239 (24%) likely contained biallelic, and 192 (19%) had most likely monoallelic *TP53*^*MT*^. Such classification was further substantiated by a survival-based model built after re-categorization. Among cases traditionally considered monoallelic, the overall survival of those with probable monoallelic mutations was similar to the one of wild-type patients and was better than that of patients with a biallelic configuration. As a result, patients with certain biallelic hits, regardless of the disease subtype (AML or MDS), had a similar prognosis. Similar results were observed when the model was applied to an external cohort. These results were recapitulated by single-cell DNA studies, which unveiled the biallelic nature of previously considered monoallelic cases.

**Conclusion:**

Our novel approach more accurately resolves *TP53* genomic configuration and uncovers genetic mosaicism for the use in the clinical setting to improve prognostic evaluation.

## Introduction

*TP53* is a pivotal tumor suppressor gene (TSG) in cancer, including myeloid neoplasia (MN). *TP53* can be affected by hypomorphic/loss-of-function (LOF) lesions occurring in diverse configurations. In addition to truncated (frameshifts and stop codons), missense mutations in various hotspots may exert dominant-negative effect^1,2^. Poor prognosis has been attributed to *TP53* mutations (*TP53*^*MT*^) and loss of heterozygosity (LOH) by 17p deletion (del17p) in MN. In particular, this is true for instances such as complex karyotype and presumed chemotherapy-related causation^3,4^. Considering the many variables of *TP53*^*MT*^ topography and configuration, the assessment of the prognostic impact of *TP53* lesions may be challenging^3,5^.

Congenital heterozygous *TP53*^*MT*^ in Li-Fraumeni syndrome (LFS) are the first hits in the oncogenic cascade preceding the evolution of cancers after biallelic inactivation of this gene, a process consistent with a 2-hit theory of recessive TSG inactivation (Knudson’s hypothesis)^6^. Incomplete penetrance in LFS indicates that the residual function of *TP53* is at least partially protective^7^. Monoallelic *TP53*^*MT*^ may also occur in clonal hematopoiesis without overt leukemia^8^. Thus, somatic monoallelic lesions might not be directly leukemogenic if not accompanied by subsequent hits in trans configuration or affecting other genes. In early SNP-arrays studies, we have shown that some patients with *TP53*^*MT*^ may harbor biallelic inactivation by somatic copy-neutral LOH (CN-LOH) or cryptic macro/microdeletions^9^. Presumed monoallelic somatic *TP53*^*MT*^ did not affect survival but biallelic hits did confer an unfavorable prognosis.^10,11^. Furthermore, second *TP53* hits represent the most associated lesions in patients with primary *TP53*^*MT*^ with usually sweeping biallelic subclones, consistent with their functional and clinical impact^12^.

The correct assessment of *TP53* inactivation status in patients with *TP53*^*MT*^ MN in a clinical setting is complicated, as the distinction between two monoallelic hits in a form of subclonal mosaicism vs. true biallelic lesions is not easily possible. Moreover, cases with seemingly monoallelic *TP53*^*MT*^ likely contain cryptic clones with biallelic *TP53* inactivation, but their detection is not possible using traditional sequencing methods. Even after estimating the clonality of del17p or uniparental disomy (UPD), such *TP53* configuration analysis is hampered by essential flaws such as the inability to: i) detect and quantify the biallelic fraction in cases with smaller variant allelic frequency (VAF) or ii) prove the presence of subclonal mosaicisms with two different *TP53* mutant clones using traditional bulk DNA sequencing methods. The accuracy of single-cell sequencing technologies although allowing for such delineation, is limited by various technical and feasibility problems. For instance, use of single-cell DNA analysis is clinically impractical and may not always be conclusive due to allelic dropout and other imprecisions^11^. Similarly, SNP-arrays, while precise, are not sensitive in detecting smaller clones (< 20%) containing deletions or CN-LOH^13^. As a result, small clones possibly constituting a significant portion of occult *TP53* biallelic inactivation are likely to be overseen.^9,11,12,14^.

Most previous reports focused on myelodysplastic syndrome (MDS), and secondary acute myeloid leukemia (sAML) have shown that *TP53*^*MT*^ affect the clinical prognostic scoring^4,11,15^. Indeed, in the Molecular International Scoring System (IPSS-M) *TP53* lesions are the most impactful on clinical outcomes and the different *TP53* genomic configuration constitutes an important variable^16^. Based on the assumption that biallelic rather than single *TP53* hits are directly leukemogenic, we hypothesized that clinical outcomes such as survival might be used to differentiate cases with biallelic, often cryptic *TP53*^*MT*^ clones (and *vice versa*). Therefore, we investigated *TP53*^*MT*^ in a fashion agnostic to the subtypes of MN by devising a new rational method able to predict the impact of these mutations on prognosis in real-life scenarios to further improve the current clinical algorithms.

## Materials And Methods

### Patient cohort

We have compiled molecular and clinical data of a meta-analytic cohort of 1010 patients with *TP53* alterations, along with 6390 *TP53*^*WT*^ cases. Data were collected from The Cleveland Clinic, (CC, n = 1357), The Munich Leukemia Laboratory (MLL, n = 1962), and publicly available data sets (Memorial Sloan Kettering Cancer Center^11^, The Cancer Genome Atlas (TCGA)^17^, The BEAT AML master trial, n = 4081^18^ (**Table S4**).

### Genetic Studies

For the data collected at CC, whole exome sequencing (WES) was performed^19–21^ on a subset of samples. Paired tumor and germline DNAs were used for WES. Data were validated using a TruSeq or Nextera platform Custom Amplicon Kit (Illumina, San Diego, CA, USA). The targeted sequencing panel is shown in **Table S5**. Variants were annotated using Annovar and filtered using an in-house bioanalytical pipeline^14,19,21^. The gene sequencing methods of publicly shared data were previously described^22,23^.

### Statistical Analyses

To identify the optimal VAF cutoff able to delineate *TP53* mutation allelic status, we utilized a statistically robust strategy of subsampling-based regression on survival data. Specifically, using R package randomForestSRC^24^, we built a survival model with VAF as a single covariate and minimum node size set to 15. (Further statistical analyses in **supplementary methods**).

## Results

### Clinical characteristics of patients’ cohort

**Figure 1:**
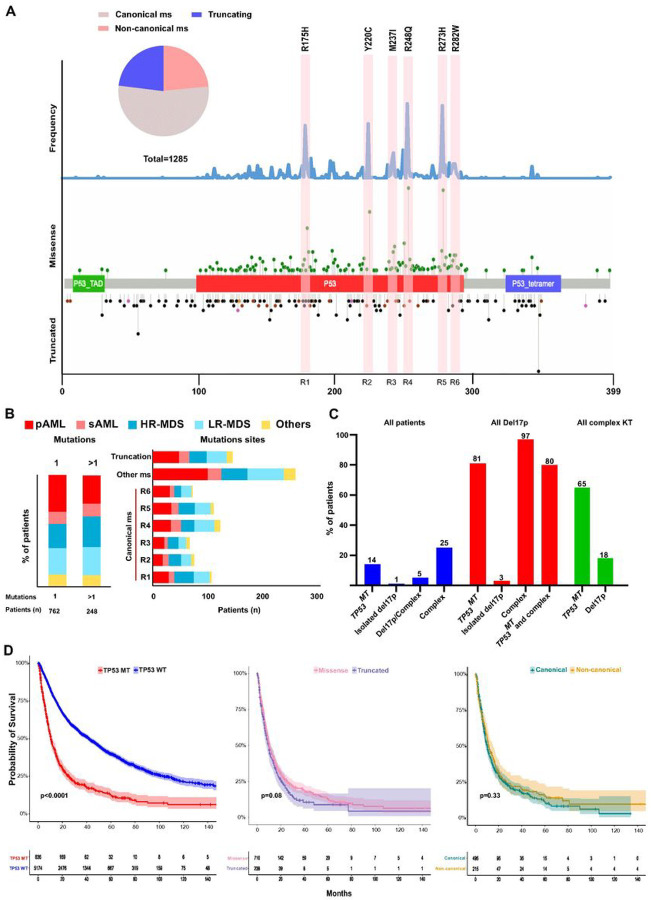
Distribution of *TP53* mutations with hotspot locations and chromosome 17 deletion. **A:** Schematic drawing of *TP53* gene showing the location of mutations, the type of mutations, and canonical sites. Frequency of mutations in all cohort is shown in the upper part. Missense and truncated mutations are indicated in the upper and lower part of the gene structure. **B:** Bar graphs showing the frequencies of single (1) and multiple *TP53* mutations (>1) and canonical missense locations in each disease subtype. **C:** Percentage of patients with cytogenetics abnormalities in relation to the *TP53* mutational status. **D:** Kaplan-Meier survival curves of patients with *TP53*mutations *vs*. wild type, missense *vs*. truncated mutations, and canonical *vs*. non-canonical missense mutations.

We screened a cohort of 7400 patients with MN and found that 1010 (14%) of the patients had 1285 *TP53*^*MT*^ (Figs. 1A & S1). The median age at diagnosis was 70 years (IQR 61–77). Low-risk MDS (LR-MDS), high-risk MDS (HR-MDS), sAML, and primary AML (pAML) were present in 34%, 17%, 4%, and 27%, respectively (Table 1**and Figure S1**). For this study, HR-MDS patients were defined by a blast count of ≥ 5%. The majority of *TP53*^*MT*^ patients harbored complex karyotype at cytogenetic evaluation (704/981; 72%) (Tables 1& **S1**).

### Distribution of TP53 mutations and 17p deletions

Of *TP53*^*MT*^ detected, missense mutations were registered in 74% of patients, truncations in 15%, while 11% had concomitant missense and truncated hits (Fig. 1A). Missense mutations were distributed into 6 main sites, including canonical hotspot lesions (defined here as ± 5 amino acids from the most canonical sites, Fig. 1A). Majority of the missense *TP53*^*MT*^ (69%) were detected in canonical sites (R175H, Y220C, M237I, R248Q, R273H, R282W)^1^**(Figure S2)**. Notably, we did not observe any differences in the number of *TP53*^*MT*^ nor in the location of the lesions among various MN subtypes (Fig. 1B). Overall, 203 patients had del17p, of whom 118 (58%) had missense *TP53*^*MT*^, and 17% had truncated *TP53*^*MT*^, 6% patients had concurrent missense and truncated *TP53*^*MT*^, while in 19% no mutation was found. Mutations often coincided with deletions of *TP53* locus either as isolated lesions or more often in the context of complex karyotype, wherein *TP53*^*MT*^ was found in 81% of patients with del17p (Fig. 1C). Irrespective of configuration, *TP53*^*MT*^ carriers had worse overall survival (OS) compared to *TP53*^*WT*^ carriers (HR 2.7 [95%CI 2.53–3.02]). No significant differences in OS between truncated and missense *TP53*^*MT*^ or canonical and non-canonical missense *TP53*^*MT*^ were observed (Figs. 1D & 1B).

### Different TP53 configurations and disease subtypes impact prognosis

**Figure 2:**
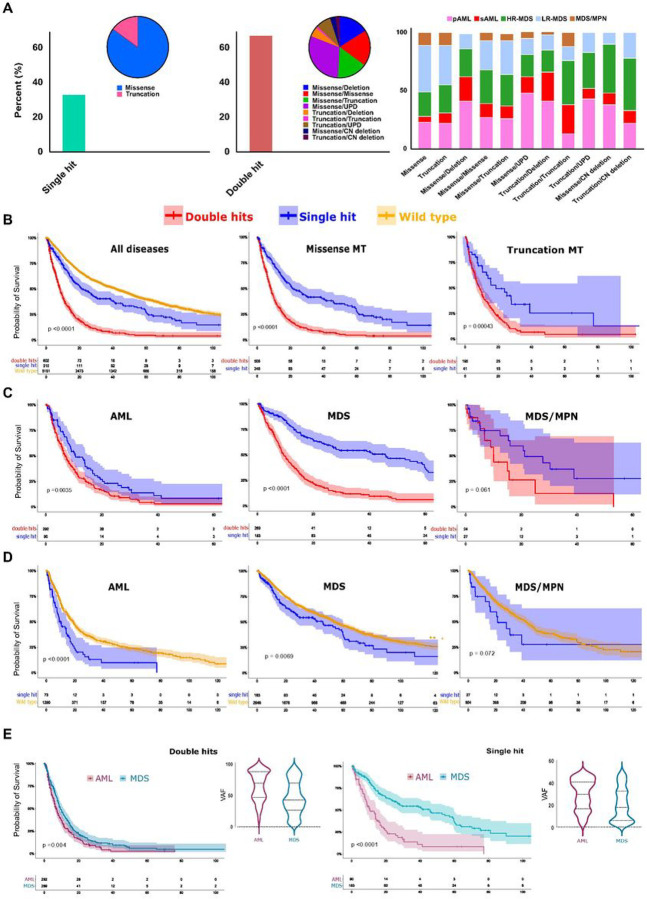
Diverse *TP53*configurations and disease subtypes impact prognosis. **A:** patients with single hit (left panel) *vs*.double hits (middle panel) *TP53* lesions and the type of configuration for each hit class and various configurations among different myeloid neoplasms (right panel). **B:** Kaplan-Meier survival estimates of patients with *TP53*wild type, single hits, and double hits, all patients (left panel) and cases with missense (middle panel) and truncated mutations (right panel). **C:**Kaplan-Meier survival estimates of patients with *TP53* single hits and double hits distributed according to different disease subtypes: AML (left panel), MDS (middle panel), and MDS/MPN (right panel). **D:** Kaplan-Meier survival estimates of patients with *TP53* wild type and single hit distributed according to different disease subtypes: AML (left panel), MDS (middle panel), and MDS/MPN (right panel). **E:** Kaplan-Meier survival estimates of *TP53*double hits (left panel) and single hit (right panel) in acute myeloid leukemia (AML) *vs*. myelodysplastic syndrome (MDS) in correlation with variant allelic frequency (VAF) for each subtype.

Following the traditional definition^10,11,25^, we categorized the patients into single- and double-*TP53*^*MT*^ hit groups. Briefly, a single *TP53* hit was defined as either: i) one *TP53* mutation or ii) isolated 17p deletion, while double *TP53* hits were defined as *TP53*^*MT*^ and: i) another *TP53*^*MT*^ or ii) 17p deletion or iii) *TP53* locus UPD. We found that 36% of *TP53*^*MT*^ patients had single hits, while 64% exhibited double hits (Fig. 2A). *TP53* double hits with different configurations were enriched in p/sAML and HR-MDS cases (Fig. 2A, middle and right panels and **Figure S3**). Overall, carriers of *TP53* double hits, whether missense and/or truncated had a worse OS than those with single *TP53* hits (HR: 2.5 [2.08–3.02], Figs. 2B & S4). Sub-group analysis according to the underlying disease morphology yielded similar results (*data not shown*). However, the significantly larger difference in OS between single and double *TP53* hits was observed in MDS (HR: 3.1 [2.43–4.09]) compared to AML (HR: 1.5 [1.16–2.04]; Fig. 2C). *TP53*^*MT*^ was also associated with worse OS when we compared *TP53* single hit to *TP53*^*WT*^ in both AML (HR: 1.8 [1.42–2.46]) and MDS (HR: 1.3 [1.08–1.67]) but not in MDS/MPN subtype (Figs. 2C and 2D). Finally, the OS of AML cases was consistently poorer compared to that of MDS patients in both single and double hits groups (Fig. 2E). Paralleling these findings, such a worse prognosis was also accompanied by a significantly higher VAF of *TP53*^*MT*^ in AML *vs*. MDS in both double hits (64% *vs*. 46.5%, *p* < 0.001) and single hit (median 30 *vs*. 18%, *p* < 0.001; Fig. 2E).

### A new approach to resolve the dilemma of biallelic vs. monoallelicTP53^MT^

**Figure 3:**
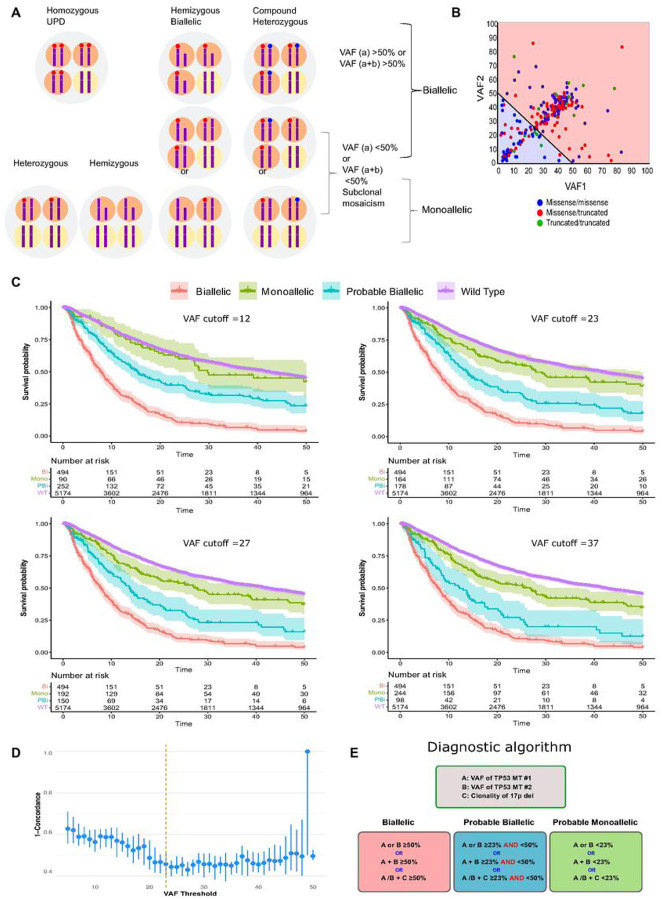
Assessment of allelic status of *TP53* lesions. **A:** possibilities of different *TP53* configurations and del(17p) presence resulting in monoallelic *vs*. biallelic lesions. For illustration purpose, four cells scheme is presented. For biallelic mutations, the sum VAF% is more than 50% in hemizygous, homozygous uniparental disomy (UPD) or compound heterozygous configurations. For monoallelic mutations, the sum VAF% is less than 50% in different configurations including heterozygous, hemizygous, or subclonal mosaicism. **B:** The variant allele frequency (VAF) of two *TP53* mutations of patients in our cohort was plotted in two colors (red area, obligatory biallelic) with the sum of VAF1 and VAF2 exceeding 50% and (blue area, non-obligatory biallelic) with sum VAF1 and VAF2 less than 50. **C:** Kaplan-Meier survival estimates of obligatory biallelic, probable biallelic, probable monoallelic, and wild-type *TP53* patients after applying different VAF cutoffs based on random forest analysis separating probable monoallelic from probable biallelic mutations. **D**. VAF cutoffs cross-validation by randomly splitting the data into test/train sets with %20/%80 ratios and calculating the Harrell’s C-index (Concordance-index) in the test set over 30 runs. A VAF of 23% resulted optimal for separating the monoallelic and biallelic *TP53* mutations. E. A novel algorithm for the precise classification of *TP53*^*MT*^ into obligatory biallelic, probable biallelic, or probable monoallelic groups.

**Figure 4:**
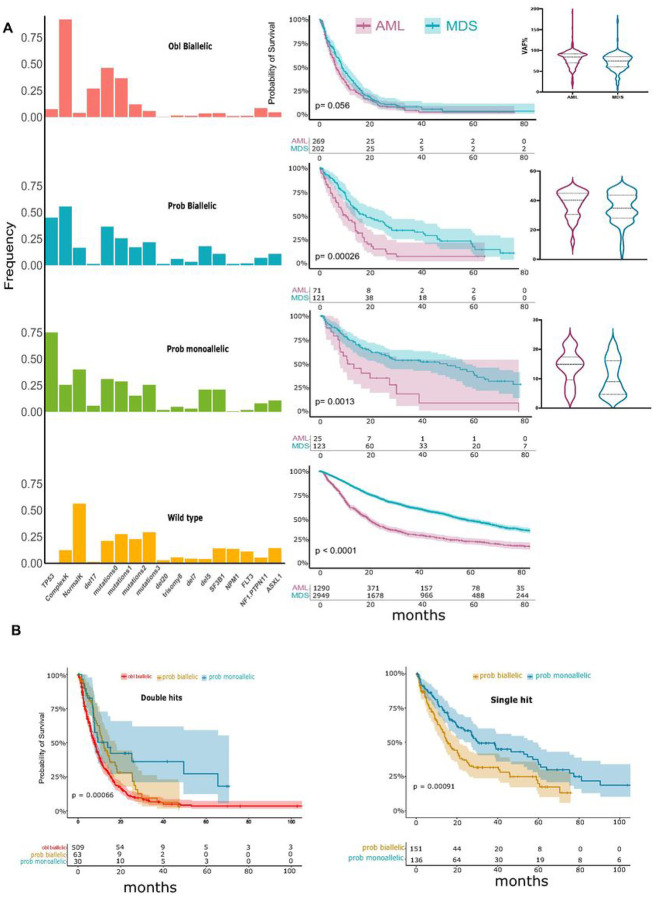
Identifying the probable allelic involvement in relation to clinical and cytogenetic factors. **A:** Frequency and importance of number of concurrent somatic mutations and cytogenetic abnormalities with Kaplan-Meier survival estimates comparing acute myeloid leukemia (AML) *vs*. myelodysplastic syndrome (MDS) for each group. Median variant allele frequency (VAF) for AML *vs*. MDS. **B:** Kaplan-Meier survival estimates comparing obligatory biallelic, probable biallelic, and probable monoallelic mutations in double hits and single hit groups.

To resolve the uncertainty regarding the precise *TP53*^*MT*^ allelic status, we developed a new approach to accurately predict the *TP53* allelic configuration. Based on VAF values available from clinical sequencing, obligatory biallelic *TP53*^*MT*^ were identified among patients with: i) one *TP53* hit and VAF > 50% or ii) two *TP53* hits with a combined VAF > 50% or iii) *TP53*^*MT*^ VAF + clonality of del17p > 50% (Figs. 3A, 3B & S6). However, the presence of subclonal mosaicism *vs*. biallelic *TP53* hits (deletion and homozygous mutation) may not be discriminated with one *TP53*^*MT*^ and VAF < 50%. Similarly, patients with ≥ 2 *TP53* hits and a combined VAF < 50% may have subclonal mosaicism of several purely monoallelic or biallelic (**Figures S7**). Previously presumed monoallelic cases by traditional classification should be differentiated into those likely true monoallelic *TP53*^*MT*^ and/or those likely containing a subclonal cryptic biallelic *TP53* lesions. Since the presence of biallelic *TP53*^*MT*^ clones is associated with poor OS, we applied a random forest regression analysis, with survival as a surrogate marker for *TP53* allelic status, to fine-tune the VAF cutoff in order to separate the questionable cases into likely monoallelic *vs*. likely biallelic. We cross-validated VAF cutoff values by randomly splitting the data into test/train sets with 20%/80% ratios and calculating the Harrell’s C-index (Concordance) in the test set over 30 runs (Fig. 3D). A VAF cutoff of 23% was found to be optimal for separating these monoallelic and biallelic *TP53*^*MT*^ (**Figures S8 &** 3D). Accordingly, we classified the *TP53*^*MT*^ into 3 main groups: A) “obligatory” biallelic, B) “probable biallelic”, and C) “probable monoallelic” groups (Fig. 3E). Based on this approach, 579 (57%) patients had obligatory biallelic *TP53*^*MT*^, 239 (24%) had probable biallelic *TP53*^*MT*^, and 192 (19%) had probable monoallelic *TP53*^*MT*^ (**Table S2**). The OS of patients with probable monoallelic *TP53*^*MT*^ (median OS: 29 [10–77]) was similar to the *TP53*^*WT*^ group (median: 42 [15–103]), *p* = 0.070. However, patients with probable biallelic *TP53*^*MT*^ (median OS: 14 [7–37]) had worse outcomes as compared to *TP53*^*WT*^(*p* < 0.001; Fig. 3C) at all VAF cutoffs. While the OS was similar when we compared AML to MDS within the obligatory biallelic *TP53*^*MT*^ group; however, AML showed a worse prognosis than comparable MDS, coinciding with higher *TP53*^*MT*^ VAFs in AML patients (Fig. 4A). Obligatory biallelic *TP53*^*MT*^ status was found to be associated with pAML (OR: 1.84, [1.44–2.96]), sAML (OR: 2.50 [1.25–4.99]), complex karyotype (OR: 10.70 [5.20–21.9]), or carriers of del17p (OR: 5.42 [2.80–10.5]) (Fig. 4A, **Table S3**).

To further highlight the limitations of the traditional classification methods, we applied our new algorithm to the previously classified single and double *TP53* hit groups. Indeed, we found significant OS differences between the new subgroups with the better prognostic resolution of the monoallelic/biallelic cases. Moreover, patients with probable biallelic *TP53* hits had survival rates between probable monoallelic *TP53*^*MT*^ and obligatory biallelic *TP53*^*MT*^ cases (Fig. 4B, **Figure S9**). This survival difference was also validated in an external confirmatory cohort (**Figure S10**).

### Confirming biallelic TP53 inactivation and clonal mosaicism in selected cases

Our novel VAF-based method allowed a re-classification of 171 (17%) patients to probable biallelic class and 34 (3.3%) patients to probable monoallelic class of *TP53*^*MT*^ compared to the traditional classification. Accordingly, VAF < 50% in single or double *TP53* hits could be indeed associated with the presence of a subclone that acquired biallelic inactivation. To further confirm these results and characterize the subclonal configurations, we selected four patients (3 likely monoallelic and one likely biallelic *TP53*^*MT*^) and applied single-cell DNA mutational and copy number analysis. We found that some cells contained monoallelic *TP53*^*MT*^ while others had biallelic lesions in all cases studied. For example, in a pAML case (UPN13), the bulk NGS showed *TP53*^*MT*^ with a VAF of 8%; however, 6% of the cells were actually biallelic. In another HR-MDS case (UPN125), the bulk NGS showed *TP53*^*MT*^ with a VAF of 33%, while 4% were biallelic. In case (UPN423), NGS detected two *TP53*^*MT*^ with a combined VAF of 20%, but this sample contained 32% biallelic cells by single-cell DNA analysis. Finally, a case (UPN875) of LR-MDS had two *TP53*^*MT*^ (missense and truncated); however, the single-cell analysis showed three *TP53*^*MT*^, and the percentage of biallelic cells was 34% (**Figure S11A-D**).

### Frequency of concurrent somatic mutations and factors associated with TP53 ^MT^

We found that 41% of cases had *TP53*^*MT*^ as a sole molecular lesion while the remaining 59% harbored additional somatic events. In particular, complex karyotype was more frequent among patients with isolated *TP53*^*MT*^ (*p* < 0.001). As to disease associations, LR-MDS cases had a lower burden of co-mutations, while the highest percentages were registered in MDS/MPN group (p = 0.0015; **Figure S12**). Interestingly, the rate of co-occurring events varied according to the *TP53*^*MT*^ status. The *TP53*^WT^ group had a rate of 2.10 co-mutation per patient. In contrast, this rate was lower in other groups with rates of 0.80, 1.42, and 1.76 co-mutations per patient in obligatory biallelic, probable biallelic, and probable monoallelic groups, respectively (**Table S3, Figure S13**). We then applied our new classification scheme to investigate whether any difference was notable in the mutational configuration between patients with obligatory *vs*. probable monoallelic and probable biallelic *TP53*^*MT*^. In univariate analysis, we found that *IDH1, IDH2, EZH2, SUZ12, ASXL1, DNMT3A, JAK2, RUNX1, SF3B1, SRSF2, TET2*, and *U2AF1* mutations were less common in obligatory biallelic patients compared to probable monoallelic/probable biallelic cases; however, this correlation was not significant in a multivariate setting (**Table S3**).

## Discussion

The prognostic impact of *TP53*^*MT*^ depends on the allelic configuration of the *TP53* hits, we reversely engineered a clinically applicable system enabling the imputation of allelic status that allows for a more precise assignment of prognosis using routinely available molecular tools. The underlying hypothesis for our strategy was that traditionally defined single hit cases^11,25^ might also contain subclones with a biallelic *TP53* inactivation, which negatively influences prognosis.

By applying a rationally developed strategy to our large and well-annotated cohort, we provide a method for a more precise assessment of prognosis in carriers of *TP53* lesions under the assumption that the higher the clonal burden, the more likely is the presence of a cryptic biallelic subclone. Using a newly devised bioinformatics approach, we established a VAF cutoff for prognostic diversification of traditionally considered monoallelic *TP53*^*MT*^ groups. Conversely, we have also shown that double hits are not necessarily biallelic but may constitute a subclonal *TP53* mosaicism in a branching evolution mode. A more intricate method such as single-cell DNA sequencing, including the simultaneous analysis of mutations and CN-LOH, demonstrated the above-described points, in agreement with the observation in LFS.

*TP53*^*MT*^ was common in MDS and AML patients in agreement with other studies^26,27^, while our earlier analyses have shown that a second *TP53* lesion was the most common hit associated with *TP53*^*MT*14^ and that second *TP53* hits are likely sweeping lesions^12^. Previous studies in MDS^11,25^ have already demonstrated that monoallelic lesions have no clinical impact on prognosis *vs. TP53*^WT^ cases, but biallelic clones defined by the presence of two *TP53* lesions have more aggressive phenotypes. We initially confirmed this finding according to traditional methods, but our current strategy, as described, further refined both double and single hit cases so that the latter could be sub-stratified according to the probability of the presence of truly biallelic subclones and thereby distinguished by worse outcomes and vice versa. For instance, patients with *TP53*^*MT*^, whether single or double hits and combined VAF of < 23% rarely harbor biallelic subclones and indeed show WT-like survival. Conversely, *TP53*^*MT*^ patients with a VAF of > 23% have more aggressive disease.

To highlight the limitations of previous traditional classifications of *TP53*^*MT*^, the International Consensus Classification of myeloid neoplasms and acute leukemias group recently published new classification schemes of MN with *TP53*^*MT*^.^28^ Although the new classifications addressed the constraints of the previous schemes, the issue of possible subclonal mosaicism vs. truly biallelic hits and the possibility of cryptic biallelic hits in in seemingly monoallelic cases remained unresolved. In our algorithm, we showed that patients with single *TP53*^*MT*^ and VAF more than 23% are acting biologically like biallelic *TP53*^*MT*^ cases. We think that *TP53* VAF is more accurate to determine the biallelic involvement than the presence of complex karyotypes unless the latter contain del17p.

According to our proposed algorithm, MDS and AML with obligatory biallelic mutations had similar survival, thus upstaging MDS irrespective of the blast count. Recent findings that patients with AML have similar OS compared to those with MDS with excess blasts^25^ overlapping with the presented results.

Monoallelic *TP53*^*MT*^ are not unimportant as they constitute the first step in establishing a dominant clone characterized by biallelic *TP53* loss. Unexpectedly, we could not differentiate the prognosis between frameshift and dominant-negative missense mutations in any of the possible configurations.

Because our method relies on parameter optimization alone, while improving previous approaches, it has clear limitations. For instance, we did not consider the clonality of the samples that these data were not readily available for all the cohorts in our analysis. However, such consideration will be necessary for future studies to estimate allelic involvement accurately.

In sum, our study demonstrates the importance of delineating the subclonal mosaicism of *TP53* mutations for modeling disease progression in MN. In addition to the genetic context, the *TP53*^*MT*^ role may also vary in different disease subtypes (e.g., AML *vs*. MDS), and individual (single hit) VAF levels can shape patients’ trajectories differently. In the future, our proposed approach could be incorporated into prognostication systems^16^, such as IPSS-M to improve its precision with regard to patients’ outcomes. Ultimately, resolution of *TP53* inactivation status may proof a valuable tool for identifying the most suitable candidates for *TP53*-targeted therapeutic strategies.

## Figures and Tables

**Table 1:** Clinical and cytogenetic characteristics of the study cohort

Characteristics	All patients N (%)	*TP53*^*MT*^ N (%)	*TP53*^*WT*^ N (%)	p-value
Number of patients	7400 (100%)	1010 (14%)	6390 (86%)	
Median age at diagnosis (IQR)	70.3 (61–77)	71 (64–77)	70 (61–77)	0.001
Male gender (%)	58%	53%	59%	0.015
Disease subtypes				
pAML	1985 (26.8%)	316 (31.2%)	1669 (26.1%)	< 0.001
sAML	272 (3.6%)	111 (10.9%)	161 (2.5%)	< 0.001
HR- MDS	1273 (17.2%)	234 (23.1 %)	1038 (16.2%)	< 0.001
LR- MDS	2558 (34.6%)	244 (24.1 %)	2314 (36.3%)	< 0.001
MDS/MPN	1312 (17.8%)	105 (10.4%)	1208 (18.9%)	< 0.001
Cytogenetic data*				
Normal	2084 (47.8%)	122 (12.4%)	1967 (58.2%)	< 0.001
Complex	1083 (24.8%)	704 (71.7%)	344 (10.1%)	< 0.001
Deletion 5q	211 (4.8%)	95 (9.6%)	116 (3.4%)	< 0.001
Deletion 7	146 (3.3%)	20 (2.0%)	126 (3.7%)	0.009
Deletion 17p	207 (4.7%)	167 (17.0%)	40 (1.1%)	< 0.001
Deletion 20q	93 (2.1%)	8 (0.8%)	85 (2.5%)	0.001
Trisomy 8	252 (5.7%)	36 (3.6%)	216 (6.3%)	0.001
Deletion Y	84 (1.9%)	10 (1.0%)	74 (2.1%)	0.018
Median Hb (IQR)	9.7 (8.5–11.1)	9.1 (8.2–10.2)	9.8 (8.6–11.2)	< 0.001
Median WBC (IQR)	6.8 (3.3–23.6)	5.1 (2.6–16.3)	7.1 (3.4–24.9)	< 0.001
Median platelet (IQR)	105 (50–208)	65 (38–120)	113 (54–220)	< 0.001

* Cytogenetics were available for total of 4,358 patients, of these 981 *TP53*^*MT*^ and 3377 *TP53*^WT^

MT: mutation, WT: wild type, IQR: interquartile range, pAML: primary acute myeloid leukemia, sAML: secondary acute myeloid leukemia, HR-MDS: high-risk myelodysplastic syndrome, LR-MDS: low-risk myelodysplastic syndrome, MDS/MPN: myelodysplastic/myeloproliferative overlap neoplasms, MPN: myeloproliferative syndrome, CMML: chronic myelomonocytic leukemia, Hb: hemoglobin, WBC: white blood cells.

## Data Availability

All the data used to support our results are available in this article. NGS data of The Cleveland Clinic cohort can be requested by contacting the corresponding author (maciejj@ccf.org).

## Key Points

- Patients with single *TP53* mutations and variant allele frequency more than 23% are acting biologically like biallelic *TP53* cases.
- Traditionally defined single hits might contain subclones with biallelic inactivation which negatively influences prognosis.
